# Which sexually active young female students are most at risk of pelvic inflammatory disease? A prospective study

**DOI:** 10.1136/sextrans-2015-052063

**Published:** 2015-06-16

**Authors:** Phillip E Hay, Sarah R Kerry, Rebecca Normansell, Paddy J Horner, Fiona Reid, Sally M Kerry, Katia Prime, Elizabeth Williams, Ian Simms, Adamma Aghaizu, Jorgen Jensen, Pippa Oakeshott

**Affiliations:** 1 Department of Genitourinary Medicine, Courtyard Clinic, St George's Hospital, London, UK; 2 Population Health Research Institute, St George's, University of London, London, UK; 3 School of Social and Community Medicine, University of Bristol, Bristol, UK; 4 Department of Primary Care & Public Health Sciences, Kings College London, London, UK; 5 Pragmatic Clinical Trials Unit, Queen Mary's, University of London, London, UK; 6 Homerton Sexual Health Services, Homerton University Hospital, London, UK; 7 Health Protection Services, Public Health England, London, UK; 8 Statens Serum Institut, Copenhagen, Denmark

**Keywords:** PELVIC INFLAMMATORY DISEASE, SEXUAL HEALTH, WOMEN

## Abstract

**Objective:**

To identify risk factors for pelvic inflammatory disease (PID) in female students.

**Methods:**

We performed a prospective study set in 11 universities and 9 further education colleges in London. In 2004–2006, 2529 sexually experienced, multiethnic, female students, mean age 20.8 years, provided self-taken vaginal samples and completed questionnaires at recruitment to the Prevention of Pelvic Infection chlamydia screening trial. After 12 months, they were followed up by questionnaire backed by medical record search and assessed for PID by blinded genitourinary medicine physicians.

**Results:**

Of 2004 (79%) participants who reported numbers of sexual partners during follow-up, 32 (1.6%, 95% CI 1.1% to 2.2%) were diagnosed with PID. The strongest predictor of PID was baseline *Chlamydia trachomatis* (relative risk (RR) 5.7, 95% CI 2.6 to 15.6). After adjustment for baseline *C. trachomatis*, significant predictors of PID were ≥2 sexual partners or a new sexual partner during follow-up (RR 4.0, 95% CI 1.8 to 8.5; RR 2.8, 95% CI 1.3 to 6.3), age <20 years (RR 3.3, 95% CI 1.5 to 7.0), recruitment from a further education college rather than a university (RR 2.6,  95% CI 1.3 to 5.3) and history at baseline of vaginal discharge (RR 2.7, 95% CI 1.2 to 5.8) or pelvic pain (RR 4.1, 95% CI 2.0 to 8.3) in the previous six months. Bacterial vaginosis and *Mycoplasma genitalium* infection were no longer significantly associated with PID after adjustment for baseline *C. trachomatis*.

**Conclusions:**

Multiple or new partners in the last 12 months, age <20 years and attending a further education college rather than a university were risk factors for PID after adjustment for baseline *C. trachomatis* infection. Sexual health education and screening programmes could be targeted at these high-risk groups.

**Trial registration number:**

(ClinicalTrials.gov NCT00115388).

## Introduction

Pelvic inflammatory disease (PID) is common, often asymptomatic, and may cause tubal infertility, ectopic pregnancy and chronic pelvic pain, but case definition lacks specificity.^1–5^ *Chlamydia trachomatis* and *Neisseria gonorrhoeae* may cause respectively 25–30%^6^ and 1–2%^7^ of PID in the UK, and *Mycoplasma genitalium*^8^ and bacterial vaginosis (BV)^9^ may have a role, but in up to 70% of PID cases no pathogens are found.^8^

Using data from the Prevention of Pelvic Infection (POPI) chlamydia screening trial,^10^ the main aim of this prospective study was to identify risk factors for PID in young female students in an education setting. In a secondary analysis, we explored whether baseline BV or *M. genitalium* might be risk factors for PID independent of baseline chlamydial infection.

## Methods

### Study population

The design of the POPI trial has been described elsewhere.^10^ Briefly, during 2004–2006, 2529 female students were recruited from 20 London universities and further education colleges. (Further education colleges take students from age 16 and offer vocational subjects such as hairdressing as well as A-levels and university entry preparation courses to a diverse group of students. Courses are usually free of charge to those under 19 years.) Students were eligible to take part if they were aged ≤27 years, female, sexually experienced, not pregnant and had not been tested for *C. trachomatis* in the previous three months. They were asked to complete a questionnaire and to provide two self-taken vaginal swabs. One swab was used for the chlamydia screening trial. The other was rolled over a glass slide for BV analysis, placed in Aptima transport medium (Gen Probe), stored at −80°C and later analysed for *M. genitalium and N. gonorrhoeae.*^8^

### Twelve-months follow-up

**Figure 1:**
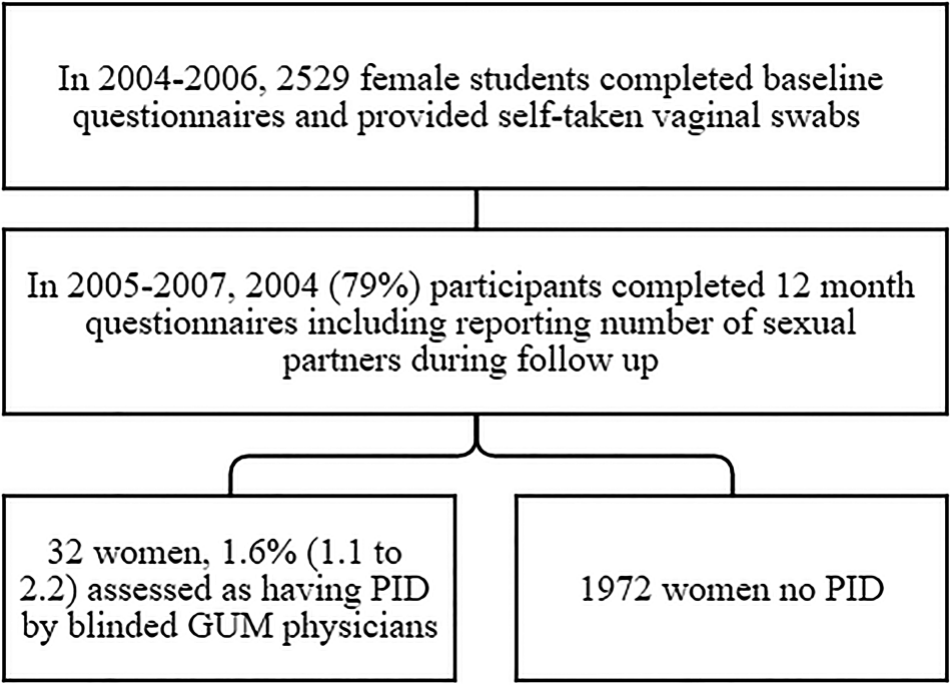
Flow of participants. GUM, genitourinary medicine; PID, pelvic inflammatory disease.

Figure 1 shows the design of this prospective study. Initial follow-up was by emailed and postal questionnaires backed by telephone calls. The follow-up questionnaire asked about development of PID or related symptoms over the past 12 months. For women with possible PID and those who did not return questionnaires, we searched for clinical details from general practitioners and/or hospital records. We also did a medical record search in 65 women who reported being diagnosed with chlamydial infection during the 12-month follow-up period.

### Diagnosis of possible PID

Since it would be unethical to demand a laparoscopic diagnosis, we used modified Hager's clinical criteria^11^ and the Centers for Disease Control and Prevention guidelines^12^ to diagnose PID. To reduce bias, confirmation of the diagnosis was done by review of all data (questionnaires backed by medical records) in a two stage process by genitourinary medicine physicians (EW, PEH and KP), resolved by PJH when there was disagreement, all of whom were blind to baseline bacteriological tests and group allocation.^10^ We included cases of PID who developed symptoms or were seen by a healthcare professional during the 12-month follow-up period.

### Statistical analysis

The sample size for this study was constrained by the 2529 women recruited to the chlamydia screening trial. As we were looking at the development of PID for which change in sexual partner may be an important behavioural risk factor,^6^ we restricted analysis to the 2004 (79%) women who answered the question on how many men they had had sexual intercourse with during the 12-month follow-up. Risk factors evaluated included age <20 years, black ethnicity, smoking, recruited at a further education college rather than a university, first sexual intercourse before age 16, two or more partners in the year prior to baseline or during 12-month follow-up, a new partner during follow-up, baseline *C. trachomatis,* BV, *M. genitalium* or *N. gonorrhoeae*; and symptoms of abnormal vaginal discharge, pelvic pain, intermenstrual bleeding or dyspareunia in the previous six months at baseline. We did not include reports of these symptoms during follow-up as a risk factor as this is part of the diagnosis of PID.^11^ ^12^

We used relative risks (RRs) to examine the relationship between previously reported risk factors^6^ ^8^ ^10^ and development of PID over 12 months. We adjusted for baseline chlamydial infection as we wanted to see whether BV or *M. genitalium* were associated with PID independent of chlamydial infection. Further adjustment for potential confounders was not carried out due to the small number of PID cases occurring during follow-up. As these data come from a trial in which 63 women in the intervention group with chlamydia at baseline were referred for treatment, we also adjusted the RRs of chlamydia causing PID for intervention or control group in a subsequent analysis. Results were presented as RRs, and adjusted risks were calculated using binominal regression.

## Results

### Follow-up

Of 2529 women recruited to the trial, 2377 (94%) were followed up after 12 months and 2004 (79%) completed the question on numbers of sexual partners during follow-up. Compared to the 2004 women with data on sexual partners who were included in the study, the 525 women not included were younger (mean age 19.9 years±2.5 vs 21.2 years±2.8, p<0.001) and more likely to be of black ethnicity: 39.8% (483/1514) vs 24.2% (205/310, p<0.001).

### Risk factors for PID

Of the 2004 women, 32 (1.6%, 95% CI 1.1% to 2.2%) were diagnosed with PID during follow-up. Table 1 shows that PID was more common in women aged <20 years, those of black ethnicity, those recruited from a further education college rather than a university, those with baseline symptoms of vaginal discharge or pelvic pain, and women with two or more sexual partners or a new partner during the 12-month follow-up. Overall, baseline infection with *C. trachomatis* was the strongest risk factor for PID (RR 5.75, 95% CI 2.63 to 12.56). Adjustment for intervention group scarcely changed this (RR 5.73, 95% CI 2.62 to 12.52).

**Table 1:** PID during 12-month follow-up, related to demographic characteristics, sexual behaviour and baseline symptoms or co-infection in 2004 women

Characteristic	% with characteristic	Cases of PID in		Relative risk (95% CI)	Relative risk adjusted for chlamydia (95% CI)
		Women with characteristic	Women without characteristic		
Age <20 years	39.9	2.84 (23/810)	0.75 (9/1194)	**3.77 (1.75 to 8.10)**	**3.21 (1.48 to 6.96)**
Black ethnicity	24.2	2.69 (13/483	1.25 (19/1514)	**2.14 (1.07 to 4.3)**	1.81 (0.87 to 3.73)
Smoker	30.4	1.81 (11/608)	1.51 (21/1392)	1.20 (0.58 to 2.47)	1.18 (0.57 to 2.44)
Further Education college versus university	26.4	3.03 (16/528)	1.08 (16/1475)	**2.79 (1.41 to 5.55)**	**2.63 (1.31 to 5.30)**
First sexual intercourse <16 years	28.4	1.61 (9/560)	1.56 (22/1392)	1.03 (0.48 to 2.23)	1.02 (0.47 to 13.10)
Two or more partners in year prior to baseline*	42.1	2.02 (17/842)	1.21 (14/1156)	1.67 (0.83 to 3.36)	1.60 (0.78 to 3.29)
Two or more partners during 12-month follow up	37.1	3.09 (23/744)	0.71 (9/1260)	**4.33 (2.01 to 9.30)**	**3.95 (1.83 to 8.50)**
New partner during 12-month follow-up	49.4	2.49 (24/965)	0.79 (8/1007)	**3.07 (1.39 to 6.82)**	**2.82 (1.27 to 6.28)**
Vaginal discharge in the past six months*	11.3	3.59 (8/223)	1.37 (24/1758)	**2.63 (1.19 to 5.78)**	**2.66 (1.22 to 5.82)**
Pelvic pain*	12.3	4.51 (11/244)	1.21 (21/1737)	**3.73 (1.82 to 7.64)**	**4.07 (2.00 to 8.28)**
Intermenstrual bleeding*	12.7	2.78 (7/252)	1.45 (25/1729)	1.92 (0.84 to 4.40)	1.90 (0.83 to 4.38)
Dyspareunia*	11.6	2.18 (5/229)	1.54 (27/1752)	1.42 (0.55 to 3.64)	1.13 (0.40 to 3.21)
Bacterial vaginosis					
BV positive	20.5	2.94 (11/374)	1.38 (20/1449)	**2.13 (1.03 to 4.41)**	1.84 (0.88 to 3.88)
BV intermediate	1.4	1.2 (1/82)	1.38 (20/1449)	0.88 (0.12 to 6.50)	0.84 (0.11 to 6.24)
Chlamydia trachomatis*	5.7	7.02 (8/114)	1.22 (23/1883)	**5.75 (2.63 to 12.56)**	
Mycoplasma genitalium*	3.2	5.08 (3/59)	1.47 (27/1811)	**3.46 (1.08 to 11.09)**	2.90 (0.89 to 9.44)
Neisseria gonorrhoeae*	0.4	0 (0/7)	1.58 (30/1901)		

Bold text shows statistically significant relative risk.

*At baseline.

BV, bacterial vaginosis; PID, pelvic inflammatory disease.

Baseline BV or infection with *M. genitalium* were also associated with PID.

### Risk factors for PID after adjustment for baseline *C. trachomatis*

Age <20 years, recruitment from a further education college, baseline symptoms of vaginal discharge or pelvic pain, and multiple or new partners during follow-up remained significant after adjustment for baseline *C. trachomatis*. The adjusted RRs for BV and *M. genitalium* were no longer significant but numbers were small, especially for *M. genitalium*.

## Discussion

### Principal findings

The strongest risk factor for PID was baseline infection with *C. trachomatis*, which increased the risk almost sixfold. After adjustment for *C. trachomatis*, independent predictors of PID were multiple or new sexual partners, age under 20 years, attending a further education college rather than a university and symptoms of vaginal discharge or pelvic pain at baseline.

### Strengths and weaknesses

This is the first UK education-based prospective study of PID. Recruitment from educational institutions in London allowed access to women not necessarily engaged with health services. Around one in four women were of black ethnicity, which is associated with poorer sexual health in the UK.^13^

The main limitation is that the clinical diagnosis of PID lacks sensitivity and specificity^3^ and we only know about symptomatic PID. This means we are likely to have missed some cases of PID and also misdiagnosed some women with PID who did not have PID. It was not possible to assess participants for PID at baseline, and therefore, the PID identified at follow-up is likely to include new cases that developed during the year (incident PID) and cases that were present at the start of the study (prevalent PID). In particular, where baseline symptoms of vaginal discharge and pelvic pain were identified as predictors of PID, for some women this may simply be reflecting an existing/prevalent case of PID.

As only 32 cases of PID were diagnosed during follow–up, multivariate analysis was not considered appropriate. Consequently, we cannot determine whether the factors identified as being associated with PID are independent of each other. For example, students at further education colleges were on average younger and more likely to be of black ethnicity than those at university, which may confound our results. Further studies would be needed to investigate independent effects within these associations.

In addition, due to the small number of PID cases, many of the adjusted RRs have wide CIs that include the possibility of both no effect and a clinically important effect. However, this data set still represents the largest education-based prospective study of PID to date.

Rates of PID may be lower in this study as half the participants were screened and treated for chlamydia as part of the clinical trial. However, this is unlikely to have influenced the associations found between PID and the other risk factors investigated. It was not possible to obtain samples at the time of PID assessment, and diagnoses were made retrospectively through follow-up questionnaires and a medical records search. Women who were excluded because they did not complete the follow-up questionnaire were younger and more likely to be of black ethnicity. However, PID rates were similar in included and excluded women.

Results may not be generalisable to different populations,^14^ particularly those not in education, employment or training (currently approximately 15% of women in England aged 16–24 years^15^). Indeed, testing in educational settings may not yield a high number of positive results for chlamydia.^16^ However, we included further education colleges and universities in socio-economically deprived areas of London, likely to have higher rates of sexually transmitted infections.

Finally, the freezing and delayed testing of samples for *M. genitalium* may have reduced sensitivity and therefore underestimated the number of baseline infections.^17^

### Comparison with other studies

As in previous reports, greater number of sexual partners and younger age were associated with PID.^14^ ^18^ ^19^ We also found a significant association between recruitment from a further education college (rather than a university) and PID. This finding may reflect both lower socio-economic status,^20^ which has been associated with PID,^18^ and the high proportion of sexually active teenagers and women of black ethnicity^10^ recruited from further education colleges. In contrast to other reports,^3^ we did not find smoking was associated with PID in our cohort.

We found an association between BV and PID, but once adjusted for baseline *C. trachomatis* infection this association was no longer significant. Evidence remains inconclusive.^9^ ^21^ ^22^ *M. genitalium* was also associated with PID before adjustment, but numbers were small with wide CIs. Previous reports^23^ ^24^ identified *M. genitalium* as an independent risk factor for PID. *M. genitalium* may be associated with fewer clinical symptoms than *C. trachomatis* and more likely to cause ‘silent’ PID.^24^

### Implications

Findings have implications for both health education and screening programmes. Further education colleges may want to consider including sexual health education as a compulsory part of the curriculum. This might encourage testing in the context of the identified risk factors, such as younger age, a change in partner and pelvic pain and/or vaginal discharge. A recent qualitative study in a further education setting found that young women value formal sexual health education and felt that current provisions are insufficient.^25^ In England, uptake of yearly opportunistic screening for *C. trachomatis* is only 24% in 16–24 year olds.^26^ While screening for chlamydia will not prevent every case of PID, these findings may encourage young women most at risk to be more aware of their sexual health, to engage in safer sex and to get tested for chlamydia after each change of sexual partner.^27^ ^28^

**Key messages**

- In this prospective study *Chlamydia trachomatis* infection was the strongest risk factor for pelvic inflammatory disease.
- Multiple or new sexual partners in the last 12 months, younger age and attending a further education college rather than a university were also predictors.
- Policymakers could consider targeting sexual health education at those with these risk factors.
